# Nonalcoholic Fatty Liver Disease and Associated Metabolic Risks of Hypertension in Type 2 Diabetes: A Cross-Sectional Community-Based Study

**DOI:** 10.1155/2017/5262560

**Published:** 2017-03-26

**Authors:** Xiaoying Ding, Ying Xu, Yufan Wang, Xiaohua Li, Chunhua Lu, Jing Su, Yuhang Ma, Yuting Chen, Yanhua Yin, Lijun Zhang, Yong Wu, Yaqiong Jin, Lijun Zheng, Songmei Xu, Xiuli Zhu, Jilin Ma, Lihua Yu, Junyi Jiang, Naisi Zhao, Qingwu Yan, Andrew S. Greenberg, Qianfang Huang, Qian Ren, Haiyan Sun, Mingyu Gu, Li Zhao, Yunhong Huang, Yijie Wu, Chunxian Qian, Yongde Peng

**Affiliations:** ^1^Department of Endocrinology and Metabolism, Shanghai General Hospital, Shanghai Jiao Tong University School of Medicine, 100 Haining Rd, Shanghai 200080, China; ^2^Obesity and Metabolism Laboratory, Jean Mayer USDA Human Nutrition Research Center on Aging, Tufts University, Boston, MA 02111, USA; ^3^Department of Internal Medicine, Sijing Hospital, 389 Sitong Rd, Shanghai 201601, China; ^4^Department of Chronic Disease Prevention and Control, Sijing Community Health Service Center of Songjiang District, 108 North Jiangchuan Rd, Shanghai 201601, China; ^5^Shanghai Pudong New Area Center for Disease Control and Prevention, 3039 Zhangyang Rd, Shanghai 200136, China; ^6^Department of Public Health and Community Medicine, Tufts University School of Medicine, Boston, MA 02111, USA

## Abstract

The mechanisms facilitating hypertension in diabetes still remain to be elucidated. Nonalcoholic fatty liver disease (NAFLD), which is a higher risk factor for insulin resistance, shares many predisposing factors with diabetes. However, little work has been performed on the pathogenesis of hypertension in type 2 diabetes (T2DM) with NAFLD. The aim of this study is to investigate the prevalence of hypertension in different glycemic statuses and to analyze relationships between NAFLD, metabolic risks, and hypertension within a large community-based population after informed written consent. A total of 9473 subjects aged over 45 years, including 1648 patients with T2DM, were enrolled in this cross-sectional study. Clinical and biochemical parameters of all participants were determined. The results suggested that the patients with prediabetes or T2DM were with higher risks to have hypertension. T2DM with NAFLD had significantly higher levels of blood pressure, triglyceride, uric acid, and HOMA-IR than those without NAFLD. Data analyses suggested that hypertriglyceridemia [OR = 1.773 (1.396, 2.251)], NAFLD [OR = 2.344 (1.736, 3.165)], hyperuricemia [OR = 1.474 (1.079, 2.012)], and insulin resistance [OR = 1.948 (1.540, 2.465)] were associated with the higher prevalence of hypertension independent of other metabolic risk factors in type 2 diabetes. Further studies are needed to focus on these associations.

## 1. Introduction

Diabetes is one of the most common metabolic disorders around the world [1]. It is estimated that diabetes affects 382 million people all over the world and this number is expected to rise to 592 million by 2035 [1]. Hypertension (HTN) is a common problem in the diabetic population with estimates suggesting a prevalence exceeding 60% [2]. During the past decade, there has been an increasing interest in the relationship between the exposure to type 2 diabetes (T2DM) and the development of hypertension (HTN). Many studies of prevalent hypertension in diabetes have been reported, and it is well established that people who have diabetes may have a higher chance of developing hypertension. Recently, epidemiological studies indicate that type 2 diabetes mellitus and concomitant hypertension are associated with high risks of macrovascular and microvascular complications as well as clinical adverse cardiovascular accident [3–5]. Although the factors such as excessive caloric intake and insulin resistance are involved in the pathogenesis of hypertension in T2DM and these have been targeted for therapeutic intervention [6], however, up to now, the mechanisms facilitating HTN in T2DM individuals are still not very clear [7]. More research studies are needed to determine the causes of HTN in T2DM.

Nonalcoholic fatty liver disease (NAFLD) has been increasing worldwide in the last decades [8, 9] and is occurring in up to 75% among patients with T2DM [10–12]. Recently, it has been suggested that NAFLD could increase the risk of insulin resistance (IR) and may be involved in the pathogenesis of cardiovascular disease in T2DM [13, 14]. However, currently, there has been scarce literature on the study of high metabolic risk of HTN in type 2 diabetes mellitus with or without NAFLD. Little is known about the relationship between NAFLD and HTN in this patient population [15], which limits the understanding of the relative crosstalk between NAFLD and the other metabolic risks contributing to prevalent HTN in T2DM. Taking into account that NAFLD is the most common chronic liver disease among patients with T2DM, studying the effects of NAFLD on the pathogenesis of HTN should be considered in T2DM with and without NAFLD separately. Therefore, based on this view, the main aims of this study were to comprehensively investigate the associations between NAFLD diagnosed on ultrasonography and the other metabolic risks of prevalent hypertension in a large sample of patients with type 2 diabetes.

## 2. Materials and Methods

### 2.1. Study Participants and Data Collection

From September 2013 to March 2014, a total of 9473 local inhabitants aged over 45 years who lived in Sijing, Shanghai, China, for at least 1 year, which represent the ten rural communities, were enrolled in this cross-sectional study after written informed consent. A standard questionnaire was administered by trained research staff to obtain demographic characteristics, history of disease, and family history of disease. Lifestyle risk factors such as smoking status and alcohol consumption were also assessed. The interview included questions related to the diagnosis and current treatment of diabetes and HTN as well as other specific diseases such as hyperuricemia, dyslipidemia, cardiovascular disease, renal failure, and hepatic cirrhosis. Through multiple screening, after exclusion of 1140 individuals for whom questionnaire, demographic information, physical examinations, or fasting plasma glucose (FPG) or postprandial glucose (PPG) were incomplete, 8333 subjects were left. Subjects who suffered from severe diseases that could significantly affect blood pressure, such as thyroid dysfunction, adrenal disorders, and chronic renal failure, were excluded. Subjects with excessive ethanol (for men, >140 g of ethanol/week; for women, >70 g of ethanol/week); infectious viral hepatitis; type 1 diabetes or other special types of diabetes; and lipid-regulating, uric acid-lowering, or insulin-sensitizing medication were also excluded. Finally, a total of 7885 subjects, including 1648 T2DM patients, were included in the final data analysis. This study was approved by the Human Research Ethics Committee of Shanghai First People's Hospital, Shanghai Jiao Tong University School of Medicine (Shanghai, China).

### 2.2. Anthropometric and Ultrasonography

All subjects were examined after overnight fasting for at least 10 h. Systolic blood pressure (SBP) and diastolic blood pressure (DBP) were measured thrice and were calculated as the mean of the three measurements. Waist circumference (WC) was measured midway between the lower costal margin and the iliac crest, and hip circumference was measured at the level of maximum extension of the buttocks. BMI was calculated as body weight in kilograms divided by body height squared in meters. Waist-hip ratio (WHR) was calculated as waist circumference divided by hip circumference. Abdominal ultrasonography was conducted in every subject with type 2 diabetes.

### 2.3. Biochemical Measurements

After at least 10 h of overnight fasting, the participants with no history of diabetes underwent the oral standard 75 g glucose tolerance test (OGTT) or standard steamed bread meal test with a self-reported history of diabetes. Plasma glucose, uric acid (UA), total cholesterol (TCH), triglyceride (TG), high-density lipoprotein cholesterol (HDL-C), low-density lipoprotein cholesterol (LDL-C), and other parameters were assessed enzymatically by an automatic biochemistry (HITACHI 7600) analyzer. Fasting plasma insulin (FINS), free T3 (FT3), free T4 (FT4), and thyroid-stimulating hormone (TSH) levels were tested using an electrochemiluminescence analyzer (Roche Cobas e 601). Glycosylated hemoglobin (HbA1c) was detected by high-performance liquid chromatography (Hemoglobin Analyzer D-10; Bio-Rad Laboratories Inc., Shanghai, China).

### 2.4. Definitions

The prediabetes and diabetes were defined according to the 2010 ADA Standards for Accessible Design criteria and classification based on FPG or PPG or on HbA1c [16]. According to the Seventh Report of the Joint National Committee (JNC-7) criteria [17], hypertension was defined as SBP  ≥ 140 mmHg, DBP  ≥ 90 mmHg, or with previously diagnosed hypertension. Overweight was defined as BMI ≥ 25 kg/m^2^, and obesity was defined as BMI  ≥ 30 kg/m^2^ according to the World Health Organization criteria [18]. Central obesity was defined as waist circumference [19] of ≥90 cm in men and ≥85 cm in women or WHR > 0.90 in men and >0.85 in women. The homoeostasis model assessments for IR index (HOMA-IR) and beta cell function (HOMA-beta) were calculated using the following formula: HOMA‐IR = fasting plasma glucose (FPG) (mM) ∗ insulin (mIU/L)/22.5; HOMA‐beta = 20 ∗ insulin (mIU/L)/(FPG (mM) − 3.5) ∗ 100%. Individuals with HOMA-IR ≥ 2.8 were considered to be insulin resistant [20]. Hyperuricemia was defined as serum UA > 420 *μ*mol/L in men and >360 *μ*mol/L in women, respectively [21]. Hypertriglyceridemia was defined as serum triglyceride levels ≥ 1.7 mmol/L [22]. Participants with fatty liver disease were diagnosed as having NAFLD after exclusion of viral hepatitis and excessive ethanol intake (for men, >20 g/day; for women, >10 g/day). NAFLD was ascertained using abdominal ultrasonography (US) that revealed a “bright” liver and a diffusely echogenic change in the liver parenchyma. US images were inspected by physicians who had no knowledge of the study objective [23]. Cigarette smoking status was defined as currently smoking more than one cigarette a day for at least 6 months. Current alcohol consumption was defined as more than 1 drink of any type per month [24].

### 2.5. Statistics

Statistical analyses were performed by utilizing SAS statistical software 9.2 (SAS Institute Inc., Cary, NC). Categorical and continuous variables were expressed as frequency (percentage) and median (interquartile range), respectively. Differences in clinical characteristics in type 2 diabetes with or without NAFLD were evaluated using *χ*^2^ test for categorical variables or a nonparametric Wilcoxon test for continuous variables. An unconditional logistic regression model was employed to estimate the adjusted odds ratios (ORs) and 95% confident intervals (CIs) of metabolic parameters with HTN status among T2DM. All statistical tests were based on two-sided probability. A *p* value less than 0.05 was considered as significant.

## 3. Results

### 3.1. Prevalence of HTN in the Total Subjects with Different Glucose Metabolic Statuses

**Figure 1:**
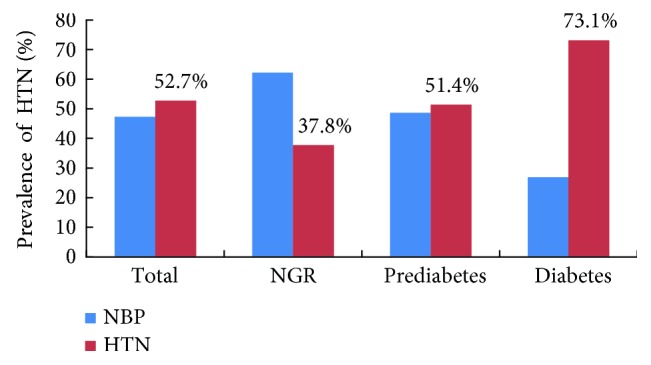
Prevalence of HTN in NGR, prediabetes, and type 2 diabetes.

To determine whether the prevalence of HTN was different in normal glucose tolerance (NGR), prediabetes, and T2DM groups, all 7885 participants over the age of 45 years were divided into the three groups according to the 2010 ADA Standards for Accessible Design criteria. The prevalence of HTN with different glycemic statuses was shown in Table 1 (Figure 1). In all the 7885 participants, the prevalence of HTN was 52.7%. However, the prevalence of HTN in patients with T2DM (*n* = 1648) was up to 73.1% which was significantly higher than 51.4% in the prediabetes group (*n* = 4046) and 37.8% in the NGR group (*p* for trend, <0.001).

### 3.2. Metabolic Characteristics of Type 2 Diabetes with or without NAFLD

A total of 1648 patients with type 2 diabetes were the subject population, out of which 686 (41.6%) were with NAFLD and 962 (58.4%) were without NAFLD. To further explore whether NAFLD and the associated metabolic parameters contribute to the pathogenesis of HTN in T2DM, all type 2 diabetes were divided into two groups according to with or without NAFLD (Table 2). There were no significant differences in the indices of gender, smoking, drinking, and antihypertensive medication between the two groups, while type 2 diabetes with NAFLD had significantly higher levels of SBP (*p* < 0.01), DBP (*p* < 0.001), waist circumference (*p* < 0.001), WHR (*p* < 0.001), BMI (*p* < 0.001), TCH (*p* < 0.001), TG (*p* < 0.001), LDL-C (*p* < 0.001), FPG (*p* < 0.001), PPG (*p* < 0.001), FINS (*p* < 0.001), HOMA-IR (*p* < 0.001), HOMA-beta (*p* < 0.001), HbA1c (*p* < 0.001), and UA (*p* < 0.001) and had significantly lower levels of HDL-C (*p* < 0.001) than that without NAFLD after being adjusted for age (Table 2).

### 3.3. Stratified Analysis of the Crosstalk between NAFLD and the Associated Metabolic Risk Factors of HTN in Type 2 Diabetes

At last, to explore whether NAFLD and the other anthropometric and metabolic parameters were independently associated with HTN, the multiple logistic regression analysis involving all the significant different parameters in T2DM with or without NAFLD, such as BMI, WHR, HOMA-IR, HOMA-beta, FPG, PPG, FINS, lipid profiles, UA, and central obesity, were performed after univariate logistic regression analysis. The subjects were divided into subgroups according to the levels of BMI, WC, WHR, lipid profiles, serum UA levels, and glycemic and NAFLD status. We next performed the stratified analysis in the subgroups (Table 3). The age-related prevalence of hypertension was 57.7%, 72.5%, 82.9%, and 84.4% among subjects aged 45 to 55 years, 55 to 65 years, 65 to 75 years, and over 75 years, respectively (Table 3). Older age was significantly associated with the prevalence of HTN. The overall prevalence of hypertension in T2DM subjects was 73.1%, while concomitant with overweight and obesity was up to 78.8% and 86.1%, respectively. Logistic regression analysis indicated that NAFLD [OR = 2.344 (1.736, 3.165)], insulin resistance [OR = 1.948 (1.540, 2.465)], central obesity [waist circumference, OR = 1.921 (1.515, 2.435); WHR, OR = 1.534 (1.185, 1.986)], overweight [OR = 2.294 (1.785, 2.948)], obesity [OR = 3.572 (2.230, 5.721)], hypertriglyceridemia [OR = 1.773 (1.396, 2.251)], and hyperuricemia [OR = 1.474 (1.079, 2.012)], but neither TCH nor LDL-C, were all significantly independently associated with the investigated presence of hypertension in type 2 diabetes after adjustment for sex, age, smoking, drinking, and family history of hypertension. In multivariate analysis, no significant relationships were found between FPG, PPG, FINS, HbA1c, FT4, TSH, and HTN in type 2 diabetes by logistic regression analysis. Additionally, the level of FT3 was also related to an increased risk of HTN in type 2 diabetes (Table 3).

## 4. Discussion

Diabetes is fast becoming a global epidemic disease. Previous studies have suggested that people with type 2 diabetes have a higher risk of developing hypertension. The prevalence of T2DM and concomitant hypertension is an increasing prototypical public health problem. Researchers have recently focused their interest on the pathogenesis of HTN in diabetes; however, the mechanisms facilitating hypertension in T2DM individuals have not been investigated thoroughly. Some recent findings suggested that insulin resistance might be involved in HTN in diabetes. Recently, extensive studies have found the role of NAFLD in insulin resistance [25–27]. Coexistence of diabetes, hypertension, and NAFLD puts people at a higher risk of developing cardiovascular problems [28, 29]. Up to now, we were unable to find any published studies to explore the role of NAFLD in pathogenesis of HTN in a large type 2 diabetes population in China.

A diagnosis of HTN often occurs immediately after a patient receives a diagnosis of diabetes. This may be because that they are more in contact with the extra screening that follows a diagnosis of diabetes. In this study, to explore whether the prevalence of HTN is different in different glycemic statuses, all participants were divided into the three groups including NGR, prediabetes, and type 2 diabetes. The trend of an increase in the prevalence of hypertension is much greater in different glycemic metabolic statuses. The prevalence of HTN in T2DM group was 73.1%, which was significantly higher than 51.4% in prediabetes and 37.8% in NGR. The prevalence of HTN in patients with prediabetes which is a high-risk state for development of diabetes was higher 1.36 times than that of NGR. It was concluded that patients with prediabetes or diabetes were with higher risk to have HTN. These emerging high cardiovascular risk patients should be the focus in community-based blood pressure management programs.

Accumulating evidence suggested a bidirectional interactive influence of NAFLD and IR [30]. IR, a key factor in the development of the metabolic syndrome, is another significant metabolic risk in the pathogenesis of HTN. NAFLD, which by itself is a risk factor for the activation of inflammation pathways, shares many predisposing metabolic risk factors with IR. In our study, to further explore whether NAFLD and the other metabolic parameters contribute to prevalent HTN in T2DM, a total of 1648 subjects with type 2 diabetes were divided into two groups according to with or without NAFLD. We found that T2DM patients with NAFLD had significantly increased levels of SBP and DBP than T2DM patients without NAFLD. Because carrying more weight in the abdomen has been linked to IR [31] and NAFLD, the waist circumference and waist-to-height ratio were also evaluated in our study. The results also suggested that the type 2 diabetes with NAFLD had significantly higher levels of waist circumference, WHR, BMI, TCH, LDL-C, FPG, PPG, FINS, HOMA-IR, HOMA-beta, and HbA1c and had significantly lower levels of HDL-C than in those without NAFLD after being adjusted for age. All these factors are established as risk factors or protective factors for HTN in T2DM.

Apart from these shared risk factors, the hyperuricemia and hypertriglyceridemia are the other proposed underlying mechanisms of insulin resistance suggested to explain the links between NAFLD and HTN [32–34]. Recently, concerns have been given to the relationships between hyperuricemia, inflammation, and development of NAFLD [35]. Furthermore, elevation of serum UA level has been identified as the predictor for NAFLD and inflammation as well as insulin resistance syndrome. Consistent with these findings, our study found that serum UA and TG levels were significantly higher in T2DM patients with NAFLD, as compared with the T2DM without NAFLD. Our findings were supported by another large population study which showed that prevalence of NAFLD was elevated in participants with hyperuricemia [36]. We tentatively put forward that hepatic disease of NAFLD and hyperuricemia may play important roles in HTN and insulin resistance by influencing the associated inflammatory pathways. Therefore, T2DM patients with NAFLD or hyperuricemia should be paid more attention to the early and the timely blood pressure management [37, 38].

Patients with insulin resistance are at increased risk of HTN in type 2 diabetes. Insulin resistance is a characteristic feature of diabetes, obesity, and NAFLD, which is characterized by the decreased tissue sensitivity to the biological effect of insulin and leading to compensatory insulin releasing. Nonetheless, there is limited data about the interaction between insulin resistance and NAFLD on the pathogenesis of HTN. In addition, IR seems to be related to the anatomical distribution of fat. Our study did find that the risk of prevalence of HTN in T2DM with central obesity according to waist circumference increased by 1.92 times than that of the controls. In the present study, in order to investigate whether the NAFLD was independently associated with the related metabolic risks, anthropometric parameters, and HTN, we next performed the stratified unconditional logistic regression analysis. The subjects were divided into subgroups according to the BMI, WC, WHR, lipid profiles; serum UA levels; glucose metabolic statuses; and NAFLD. Logistic regression analysis indicated that NAFLD, hyperuricemia, central obesity, hypertriglyceridemia, and older age, as well as insulin resistance were all significantly independently associated with the investigated presence of HTN in type 2 diabetes after adjustment for sex, age, cigarette smoking, and drinking.

The literature about the relationship between NAFLD, hyperuricemia, and HTN remains scant. In our study, the level of serum UA was the factor most strongly and independently correlated with hypertension in T2DM. The risks of prevalence of HTN increased by 1.474 times with hyperuricemia and 2.344 times with NAFLD in type 2 diabetes, respectively. These results indicated that there was a need for better understanding of the crosstalk effects between NAFLD, hyperuricemia, and HTN in diabetes. Two very recent studies reported [39, 40] that the higher free thyroxine levels were associated with a decreased risk of NAFLD. In our study, the higher T3 levels were related to an increased risk of HTN in type 2 diabetes. Taken together, these results suggested that FT3 was associated with an increased HTN risk independent of NAFLD in type 2 diabetes.

Hence, our results suggested that NAFLD, hyperuricemia, and insulin resistance could interact to concomitantly influence hypertension status and their combinations are most strongly associated with HTN in T2DM. Given these findings, prospective studies are required to clarify the causal associations of NAFLD and UA with HTN in type 2 diabetes. Therefore, in type 2 diabetes, combined intervention involving lipid-adjusting, uric acid-lowering, and insulin-sensitizing medication will more effectively lead to improvement in blood pressure, NAFLD, and glucose metabolism than either intervention alone.

Our study should be evaluated with some limitations. First, its cross-sectional design provided weak evidence of causality between NAFLD and associated risk factors of HTN in T2DM. Furthermore, the results of single-center study cannot be generalized to the overall Chinese T2DM population. Therefore, although we found that NAFLD, IR, and hypertriglyceridemia as well as hyperuricemia are associated with a higher prevalence of hypertension independent of the other metabolic risk factors in T2DM, it is still hard to be sure of any causal direction. Furthermore, multicenter cross-sectional and prospective cohort studies were needed to validate the associations.

## 5. Conclusion

We investigate the prevalence of hypertension in type 2 diabetes with or without NAFLD and examine whether NAFLD and the associated metabolic risk factors contribute to the pathogenesis of HTN in T2DM. The prediabetes and T2DM subjects had a higher prevalence of HTN. IR, TG, and UA as well as NAFLD might be independent risk factors for HTN in T2DM. These data suggested that the associations between NAFLD, HTN, IR, and hyperuricemia in T2DM are likely to be bidirectional. In conclusion, we described that NAFLD play an important role in the pathological mechanisms of HTN in T2DM. The reasonable management of NAFLD aimed to adjust hyperuricemia, hypertriglyceridemia, and IR might be beneficial in the pathogenesis of hypertension in type 2 diabetes. Further long-term cohort multicenter studies on a large scale will be necessary to verify and clarify the findings in this study in the future.

## Figures and Tables

**Table 1:** The prevalence of hypertension in the total participants including NGR, prediabetes, and type 2 diabetes.

Groups	Total subjects (*n* = 7885)		*χ* ^2^	*p* for trend
	NBP	HTN		
Total	3732 (47.3)	4153 (52.7)	443.975	<0.001
NGR	1153 (62.2)	700 (37.8)		
Prediabetes	1966 (48.6)	2080 (51.4)		
T2DM	443 (26.9)	1205 (73.1)		

NBP, normal BP; HTN, hypertension; NGR, normal glucose tolerance; and T2DM, type 2 diabetes.

**Table 2:** General demographic and metabolic characteristics of type 2 diabetes, grouped according to with or without NAFLD.

Parameters	Type 2 diabetes (*n* = 1648)		Statistic^∗^	*p* value^$^
	Without NAFLD	With NAFLD		
Participants, *n* (%)	962 (58.4)	686 (41.6)		
Gender, M, *n* (%)	482 (50.1)	328 (47.8)	0.727	0.394
Smoking, *n* (%)	249 (25.9)	163 (23.8)	0.860	0.354
Drinking, *n* (%)	138 (14.3)	120 (17.5)	3.399	0.065
Antihypertensive medication, *n* (%)	256 (26.6)	198 (28.9)	2.575	0.107
Age (years)	63.7 (56.9, 71)	59.8 (53.3, 65.2)	−8.429	<0.001
Waist circumference (cm)	86 (80, 92)	93 (88, 99)	13.767	<0.001
WHR	0.9 (0.9, 1.0)	0.9 (0.9, 1.2)	7.579	<0.001
BMI (kg/m^2^)	24.2 (22.2, 26.3)	27.2 (25.4, 29.1)	17.856	<0.001
SBP (mmHg)	142.0 (130.0, 154.7)	144.5 (133.0, 156.7)	2.767	0.006
DBP (mmHg)	77.1 (70.7, 83.3)	81.6 (73.8, 86.0)	9.009	<0.001
TCH (mmol/L)	5.0 (4.5, 5.7)	5.4 (4.8, 6.1)	6.849	<0.001
TG (mmol/L)	1.3 (0.9, 1.9)	2.1 (1.5, 3.1)	16.634	<0.001
HDL-C (mmol/L)	1.5 (1.3, 1.8)	1.4 (1.2, 1.6)	−8.940	<0.001
LDL-C (mmol/L)	2.8 (2.3, 3.4)	3.1 (2.6, 3.7)	7.803	<0.001
FPG (mmol/L)	7.1 (6.2, 8.1)	7.4 (6.6, 8.7)	5.549	<0.001
PPG (mmol/L)	13.1 (11.2, 16.5)	14 (11.7, 17.6)	3.815	<0.001
HbA1c (%)	6.2 (5.7, 7.0)	6.6 (6, 7.4)	6.849	<0.001
FINS (mIU/L)	7.1 (4.6, 10.5)	11.9 (8.6, 17.1)	17.378	<0.001
HOMA-IR	2.3 (1.4, 3.5)	4.2 (2.8, 6.2)	17.357	<0.001
HOMA-beta	39.2 (23.3, 61.9)	58.4 (39.3, 88)	11.403	<0.001
UA (*μ*mol/L)	309 (254, 366)	340 (293, 396)	7.771	<0.001

Missing values were excluded.

^∗^Chi-square value for *χ*^2^ test or *Z* value for the nonparametric Wilcoxon test.

^$^ *χ* ^2^ test for categorical variables or the nonparametric Wilcoxon test for continuous variables; *p* adjusted for age.

NAFLD, nonalcoholic fatty liver disease; DM, diabetes mellitus; M, male; WHR, waist-hip ratio; BMI, body mass index; SBP, systolic blood pressure; DBP, diastolic blood pressure; TCH, total cholesterol; TG, triglyceride; HDL-C, high-density lipoprotein cholesterol; LDL-C, low-density lipoprotein cholesterol; FPG, fasting plasma glucose; PPG, postprandial plasma glucose; HbA1c, hemoglobin A1c; FINS, fasting plasma insulin; HOMA-IR, homeostasis model assessment for insulin resistance index; HOMA-beta, homeostasis model assessment for beta cell function; and UA, uric acid.

**Table 3:** Stratified analysis of NAFLD and the associated metabolic risk factors in type 2 diabetes with or without HTN.

Parameters	Type 2 diabetes (*N* = 1648)		OR (95% CI)^∗^
	NBP (*n* = 443)	HTN (*n* = 1205)	
*Age (years)*			
45–55	160 (42.2)	218 (57.7)	1
55–65	182 (27.5)	479 (72.5)	**1.930 (1.470**, **2.533)**
65–75	68 (17.1)	330 (82.9)	**3.548 (2.522**, **4.992)**
≥75	33 (15.6)	178 (84.4)	**3.852 (2.483**, **5.977)**

*Anthropometric parameters*			
Waist circumference (cm)			
<90 M, <85 F	224 (34.4)	427 (65.6)	1
≥90 M, ≥85 F	207 (22.0)	736 (78.1)	**1.921 (1.515**, **2.435)**
WHR			
<0.90 M, <0.85 F	138 (33.8)	270 (66.2)	1
≥0.90 M, ≥0.85 F	305 (24.6)	935 (75.4)	**1.534 (1.185**, **1.986)**
BMI (kg/m^2^)			
<25.0	250 (35.0)	465 (65.0)	1
25.0–30.0	153 (21.2)	569 (78.8)	**2.294 (1.785**, **2.948)**
≥30.0	24 (14.0)	148 (86.1)	**3.572 (2.230**, **5.721)**

*Metabolic parameters*			
HOMA-IR			
<2.8	260 (33.0)	529 (67.1)	1
≥2.8	183 (21.3)	676 (78.7)	**1.948 (1.540**, **2.465)**
HOMA-beta	39.9 (22.8, 63.7)	49.8 (30.5, 77.6)	**1.010 (1.006**, **1.013)**
FPG (mmol/L)	7.1 (6.3, 8.2)	7.2 (6.4, 8.4)	1.007 (0.960, 1.057)
PPG (mmol/L)	13.4 (11.3, 17)	13.5 (11.4, 17)	0.996 (0.974, 1.020)
FINS (mIU/L)	7.3 (4.7, 11.4)	9.4 (6.2, 14)	1.008 (0.998, 1.019)
HbA1c (%)	6.3 (5.8, 7.2)	6.4 (5.9, 7.2)	1.014 (0.936, 1.098)
TCH (mmol/L)			
<4.5	92 (24.8)	279 (75.2)	1
≥4.5	351 (27.5)	925 (72.5)	0.843 (0.634, 1.121)
TG (mmol/L)			
<1.7	277 (30.3)	638 (69.7)	1
≥1.7	166 (22.7)	566 (77.3)	**1.773 (1.396**, **2.251)**
HDL-C (mmol/L)			
<1.04	39 (24.5)	120 (75.5)	
≥1.04	404 (47.8)	1084 (72.9)	0.742 (0.500, 1.102)
LDL-C (mmol/L)			
<2.6	149 (27.6)	391 (72.4)	1
2.6–3.4	172 (26.3)	482 (73.7)	1.115 (0.851, 1.461)
≥3.4	122 (26.9)	331 (73.1)	1.104 (0.818, 1.491)
UA (*μ*mol/L)			
≤420 M, ≤360 F	375 (28.7)	934 (71.4)	1
>420 M, >360 F	68 (20.1)	270 (79.9)	**1.474 (1.079**, **2.012)**

*Thyroid function*			
FT3 (pmol/L)	4.9 (4.5, 5.4)	5.0 (4.5, 5.4)	**1.211 (1.020**, **1.437)**
FT4 (pmol/L)	15.8 (14.4, 17.2)	15.8 (14.5, 17.4)	1.019 (0.973, 1.067)
TSH (mIU/L)	2.1 (1.4, 3.0)	2.2 (1.6, 3.1)	0.995 (0.966, 1.024)

*Liver disease*			
Without NAFLD	296 (37.5)	494 (62.5)	1
With NAFLD	147 (21.5)	536 (78.5)	**2.344 (1.736**, **3.165)**

^∗^Adjustment of sex, age, smoke, drinking, and family history of hypertension.

Bold values were used to indicate the significant results (*p* < 0.05).

NBP, normal BP; HTN, hypertension; M, male; F, female; WHR, waist-hip ratio; BMI, body mass index; HOMA-IR, homeostasis model assessment for insulin resistance index; HOMA-beta, homeostasis model assessment for beta cell function; FPG, fasting plasma glucose; PPG, postprandial plasma glucose; FINS, fasting plasma insulin; HbA1c, hemoglobin A1c; TCH, total cholesterol; TG, triglyceride; HDL-C, high-density lipoprotein cholesterol; LDL-C, low-density lipoprotein cholesterol; UA, uric acid; FT3, free triiodothyronine; FT4, free thyroxine; TSH, thyroid-stimulating hormone; and NAFLD, nonalcoholic fatty liver disease.

## Abbreviations

BMI: Body mass index
WC: Waist circumference
WHR: Waist-hip ratio
SBP: Systolic blood pressure
DBP: Diastolic blood pressure
TCH: Total cholesterol
TG: Triglyceride
HDL-C: High-density lipoprotein cholesterol
LDL-C: Low-density lipoprotein cholesterol
FPG: Fasting plasma glucose
PPG: Postprandial glucose
FINS: Fasting insulin
HbA1c: Hemoglobin A1c
HOMA-IR: Homeostasis model assessment for insulin resistance index
HOMA-beta: Homeostasis model assessment for beta cell function
UA: Uric acid
FT3: Free T3
FT4: Free T4
TSH: Thyroid-stimulating hormone
NAFLD: Nonalcoholic fatty liver disease.
